# GUARDING AGAINST HARM: UNDERSTANDING AND ADDRESSING ELDER MISTREATMENT RISKS IN THE PARKINSON’S POPULATION

**DOI:** 10.1093/geroni/igae098.0266

**Published:** 2024-12-31

**Authors:** Adrianne Smiley

**Affiliations:** University of Pittsburgh, Pittsburgh, Pennsylvania, United States

## Abstract

Parkinson’s disease (PD), particularly in moderate-to-advanced stages, poses significant challenges, including cognitive and physiological decline, leading to reduced independence in daily activities. Functional dependence—to any degree—is a primary predictor of mistreatment. Despite the recognized predictors of elder mistreatment risks associated with functional impairment, the understanding of its impact within the PD community remains limited. PD research often focuses on physiological changes and pharmacological and rehabilitative interventions; however, the risks for mistreatment are often unaddressed. As such, this project centers on advancing and disseminating knowledge about abuse and mistreatment within the PD population. An evidence-based, community-engaged, participatory action research approach with collaborative analysis methodology was employed with a primary focus on PD-specific organizational partnership, intra-organizational structural and procedural analysis, interdisciplinary collaboration, and relationship-building for reflection on themes, patterns, gaps, and needs for improving patient safety resources as a primary prevention method for reducing risks for mistreatment in the PD community. Outcomes: This action-based approach resulted in 1) the development of a survey instrument to gauge knowledge gaps about mistreatment in the PD community and inform the development of educational offerings for healthcare recipients, caregivers, and licensed medical providers, 2) the development of a patient safety research staffer responsible for analyzing reports submitted to the organization’s helpline to assess the prevalence of mistreatment risks according to self or caregiver reported data, and 3) establishment of a research agenda and population-centered plan for advancing knowledge about PD and elder mistreatment risks for harm prevention.

